# Acute Myeloid Leukaemia in Its Niche: the Bone Marrow Microenvironment in Acute Myeloid Leukaemia

**DOI:** 10.1007/s11912-020-0885-0

**Published:** 2020-02-11

**Authors:** E. E. Ladikou, H. Sivaloganathan, A. Pepper, T. Chevassut

**Affiliations:** 1 Brighton and Sussex Medical School, University of Sussex, Brighton, BN1 9PS UK; 2 Royal Sussex County Hospital, Brighton, BN2 5BE UK

**Keywords:** AML, Niche, Microenvironment, Bone marrow, Stromal cells, CXCR4, CXCL12, Acute myeloid leukaemia, DNMT3A, Leukaemic stem cell, Blast, Mesenchymal cells, T cells, Stroma

## Abstract

**Purpose of Review:**

Acute myeloid leukaemia (AML) is a heterogeneous malignancy for which treatment options remain suboptimal. It is clear that a greater understanding of the biology of the AML niche will enable new therapeutic strategies to be developed in order to improve treatment outcomes for patients.

**Recent Findings:**

Recent evidence has highlighted the importance of the bone marrow microenvironment in protecting leukaemia cells, and in particular leukaemic stem cells from chemotherapy-induced cell death. This includes mesenchymal stem cells supporting growth and preventing apoptosis, and altered action and secretion profiles of other niche components including adipocytes, endothelial cells and T cells.

**Summary:**

Here, we provide a detailed overview of the current understanding of the AML bone marrow microenvironment. Clinical trials of agents that mobilise leukaemic stem cells from the bone marrow are currently ongoing and show early promise. Future challenges will involve combining these novel therapies targeted at the AML niche with conventional chemotherapy treatment.

## Introduction

Acute myeloid leukaemia (AML) remains a therapeutic challenge due to its heterogeneity. It is characterized by uncontrolled expansion of myeloid progenitors in the bone marrow (BM) and the peripheral blood. Approximately 80% of patients undergo complete remission, according to the AML10 study. However, the long-term disease-free survival at 6 years was shown to be only 40%, mainly due to relapse risk which was estimated as high as 50% [1]. Patients with AML have a 10% 5-year overall survival from first relapse. Most patients do not achieve a second remission and, as a result, do not have an opportunity for a potential cure [2]. Relapse after initial response to chemotherapy remains a challenge. New therapeutic strategies are needed, focusing on the elimination of the remnant chemo-resistant leukaemic cells in the bone marrow, thus preventing relapses.

Several different treatment modalities are currently used in AML, including intensive chemotherapy (induction, consolidation, maintenance or palliative), treatment with hypomethylating agents (i.e. Azacytidine), haematopoietic stem cell transplantation (HSCT) and best supportive care. Age, gene mutations and cytogenetics of the leukaemic clone are known to drive leukemogenesis and are important prognostic factors. To date, approximately 30 gene mutations have been identified affecting prognosis in AML, the most important being: FMS-like tyrosine kinase 3 (FLT3), nucleophosmin 1 (NPM1), DNA methyltransferase 3A (DNMT3A), tumour protein 53 (TP53), TET methylcytosine dioxygenase 2 (TET2) and isocitrate dehydrogenase (IDH1/2) [3].

## Leukemogenesis

The bone marrow is a viscous tissue within the bone, which is primarily responsible for haematopoiesis. The concept of the specialised niches was originally described in 1978 by Schofield [4]. Two main anatomical BM niches have been described: the vascular and the endosteal niches, which are closely related and work collaboratively [5]. The interactions include several cytokines, the extracellular matrix, adhesion factors, which affect colonization, differentiation, and homing of haematopoetic stem cells (HSCs) [6]. The endosteum is located close to trabecular or cortical bone and is lined by osteoblasts (OB) and osteoclasts (OC). The perivascular niche is located close to sinusoids and arterioles, including the surrounding supportive stromal cells and extracellular matrix (ECM) [7, 8]. In reality, the BM microenvironment is dynamic and separation of the two BM niches is difficult, as HSCs interact with numerous structures and through several signalling axes simultaneously [8].

Haematopoietic stem cells are primitive multipotent stem cells initially formed during embryogenesis. They then move to foetal spleen and liver, and finally migrate to the BM, where they reside in separate specialised niches. The HSCs remain in the BM until maturation, where they interact with other surrounding supporting cells such as sympathetic neurons, extracellular matrix, arteriolar and sinusoidal endothelial cells and BM stromal cells (also known as mesenchymal stem cells (MSC)), which can differentiate into osteoblasts, chondrocytes, fibroblasts and adipocytes [9]. The interaction between the niche and HSC regulates their function and properties such as proliferation, differentiation, localization and self-renewal. During normal myelopoiesis, HSCs differentiate into mature blood cells via progenitor populations, of which there are mainly two lineages: the common lymphoid progenitor (CLP) and the common myeloid progenitor (CMP).

During the early stages of myeloid malignancies, the HSCs may accumulate genetic mutations that transform them into leukaemic stem cells (LSCs). The LSCs can then remodel the niche into a favourable environment for expansion or can even induce leukaemic transformation. Alternatively, mutations may occur in the progenitor populations such as lymphoid-primed multipotent progenitors (LMP) and granulocyte macrophage progenitors (GMP) [10]. Later in the disease, the LSCs become independent of the BM signals and localize more centrally. The stromal cells acquire an abnormal phenotype, and angiogenesis increases [11]. The aim of chemotherapy is to eradicate the LSC population, but at the same time, it damages the other cells of the niche, triggering regeneration. Prolonged treatment induces the development of resistance mechanisms, some of which are mediated by stromal or endothelial cells and results in LSCs which persist after chemotherapy and mediate disease relapse [11].

## Interactions Within the BM Microenvironment

**Fig. 1:**
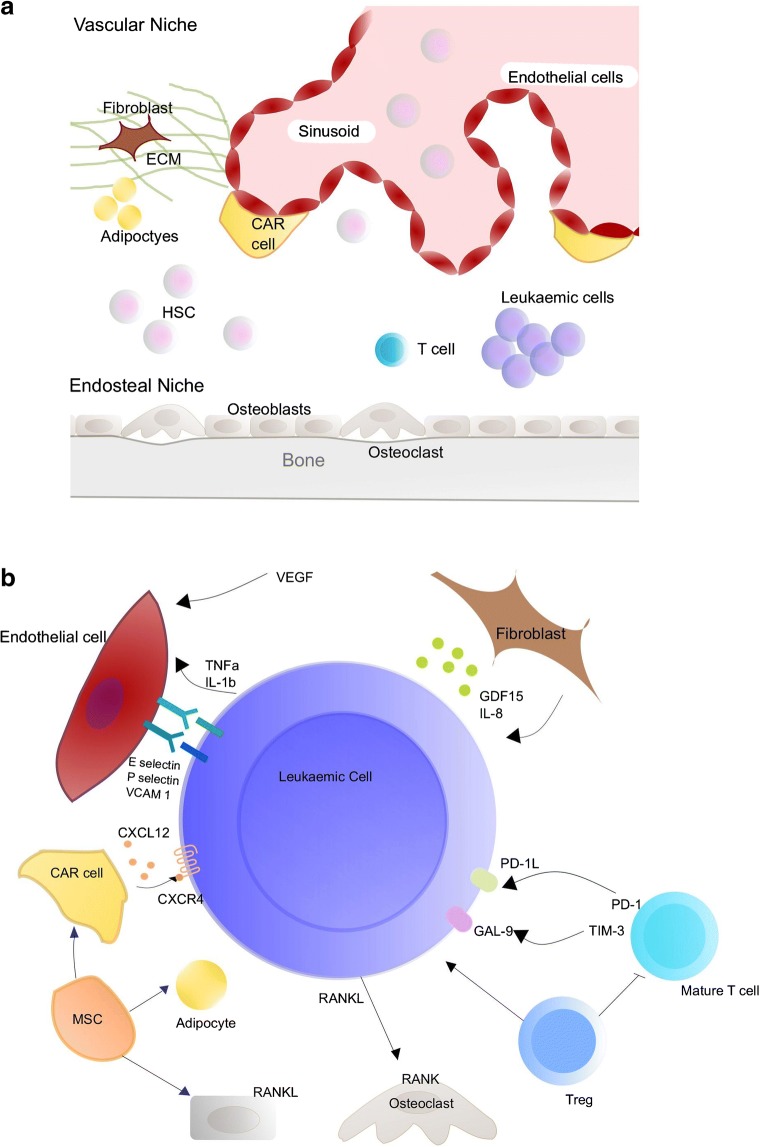
**a** Components and structure of the haematopoietic niche. **b** Summary of interactions between AML cells and the bone marrow microenvironment

The mechanism of stroma-mediated protection of leukaemic cells is complex and involves several cytokines, chemokines and adhesion molecules produced by the surrounding BM non-haemopoietic cells. The AML cells, their neighbouring stromal cells, normal haematopoietic cells as well as the infiltrating immunocompetent cells in the bone marrow produce survival and growth-regulatory chemokines and express a wide range of chemokine receptors. There are two major chemokine subclasses, CCL and CXCL chemokines, which interact with CCR and CXCR membrane receptors, respectively [12]. Several cytokines, chemokines and other soluble factors have been implicated in the AML-BM niche bidirectional crosstalk including CXCR2, CXCR4, IL6R, LFA, VLA4, RANK and FAT/CD36 [9]. A few of the interactions are summarized in the figure below (Fig. 1).

Bruserud et al. proposed a classification system of AML patients into distinct subsets according to their chemokine responsiveness and chemokine release profile. Three chemokine release groups were identified by the cluster and principal component analyses using 68 AML patients and 22 chemokines: (i) CCL2-4/CXCL1/8, (ii) CCL5/CXCL9-11, and (iii) CCL13/17/22/24/CXCL5. AML cells from patients without detectable chemokines were shown to have decreased chemotaxis. No correlations were found between any patient cluster and cytogenetic abnormalities [12].

## Endothelial Cells

Endothelial cells are located within the BM sinusoid. Their main function is to regulate the migration of cells between the BM and circulation by providing a vascular network [13]. Leukaemic cells secrete cytokines, in particular TNF-alpha and IL-1 beta, which in combination with the direct contact between adhesion receptors activate endothelial cells. As a result, leukaemic cells appear to promote their own adhesion to vascular endothelium [14]. The main pathways through which myeloblasts adhere to human endothelial cells are via E-selectin, vascular cell adhesion molecule-1 (VCAM-1) [15] as well as P-selectin. HSCs express P-selectin glycoprotein ligand-1 (or CD162), CD44 and E-selectin receptors as well as Very late antigen 4 (VLA-4), which is an α4β1 integrin that facilitates the adhesion of AML cells to VCAM-1 and fibronectin [9]. Other receptors for VCAM-1 are α4β717 and α9β1, which have also been shown to be involved in the interaction between HSC and the BM niche [16]. P- and E-selectin mediate the initial rolling of HSC on the endothelium and once slowed, HSC can then adhere via integrins and migrate to BM stroma through the endothelium [16]. Hyaluronic acid (HA) is the major ligand of CD44 and comprises an important component of the ECM in many organs, including the BM, where it is produced by endothelial, stromal and haematopoietic cells [17]. Ellis et al. showed that transplanted HSC home preferentially to the trabecular-rich metaphysis of the femurs, where they exist in close association with blood vessel endothelial cells that express high levels of HA. This confirms that HA is fundamental for the homing of HSCs to the metaphysis [18]. The interactions between AML and endothelial cells promote angiogenesis through the Notch/Dll4 pathway [19]. The proangiogenic factor vascular endothelial growth factor (VEGF) was found to be high in AML patients leading to angiogenesis and decreased rate of apoptosis. Finally, culturing endothelial cells with VEGF induced an increase of granulocyte-macrophage colony-stimulating factor (GM-CSF) secretion by endothelial cells, which is known to stimulate growth in AML cells [20].

## Fibroblasts

Fibroblasts are important in the survival and migration of leukaemic cells. Runingen et al. cultured primary AML blasts with normal human BM stromal cells and two fibroblast lines separated by a semipermeable membrane, which resulted in enhanced proliferation, antiapoptotic signalling and increased levels of proangiogenic IL8 [21]. Zhai et al. demonstrated that there are several functional cancer-associated fibroblasts (CAFs) within the BM of AML patients. These produce growth differentiation factor 15 (GDF15), which protects the AML cells from chemotherapy [22].

## Osteoclasts and Osteoblasts

The endosteum is lined by osteoblasts and osteoclasts, which regulate bone formation and resorption and form an important part of the bone marrow microenvironment. Multiple myeloma cells adhere on BM stromal cells, which induce osteoclastogenesis [23]. The main pathways involved in increased osteoclast formation in multiple myeloma are osteoprotegerin (OPG), macrophage inflammatory protein (MIP)-1α, IL-6, IL-3 and RANK ligand (RANKL). The RANK/RANKL pathway is critical for bone remodelling. RANK is a TNF receptor found on the surface of osteoclasts, and its ligand RANKL is a membrane-bound protein found on stromal cells and osteoblasts, and is also secreted by activated lymphocytes. Their binding induces osteoclastogenesis and increases osteoclast survival [24]. RANK is also expressed on natural killer (NK) cells. RANKL is expressed in AML patient cells and the factors elicited by RANK/RANKL signalling induce RANK on NK cells and impair their antileukaemic activity [25]. The endosteal surface is high in calcium ion concentration, which attracts and retains HSC through their calcium-sensing receptor (CaR) in vivo [26].

Osteoblasts form an interface between the marrow and the bone and can be divided into two types: spindle-shaped N-cadherin + osteoblasts (SNO) and oval-shaped osteoblasts [27]. Battula et al. showed that AML cells induce osteogenic differentiation in MSC to support leukemogenesis. In fact, AML cells were shown to promote osteoblastic and inhibit adipogenic differentiation of MSCs, suggesting that a AML cells enhance their expansion by inducing a preosteoblast-rich niche in the BM [28•]. Using an immunocompetent in vivo model of AML, Frisch et al. showed that leukaemia decreases osteoblasts and induces bone loss via reduced levels of osteocalcin [29]. Osteopontin is an extracellular matrix protein produced by osteoblasts and osteoclasts, which has been shown to mediate HSC localization to the endosteal niche. Osteoblasts were shown to regulate the HSC niche through Notch 1 activation on HSC, which increased their expansion in vivo [30]. Zhang et al. confirmed this finding using a different model of mutant mice, where increased osteoblasts led to increased HSC number. BMP signalling was shown to control the number of HSCs by regulating niche size [27]. The expression of tyrosine kinase Tie2 receptor on HSC was associated with quiescent HSCs, and its interaction with its ligand angiopoietin-1 (Ang-1) enhanced adhesion to osteoblasts through activation of β1-integrin and N-cadherin [31]. Published literature suggests that loss of CXCL12 expression by osteoblast is a key step in cytokine-induced HSC mobilization [32]. The CXCL12/CXCR4 axis is fundamental for the HSC and LSC interaction with the BM microenvironment and will be discussed in detail below.

## Adipocytes

Adipose tissue, in particular its stromal vascular fraction (SVF), was shown to act as an extramedullary reservoir for haematopoietic stem and progenitor cells [33]. In adult humans, the adipose tissue accounts for up to 70% of the bone marrow. The role of adipocytes remains controversial. Adipocytes were implicated as predominantly negative regulators of the BM micro-environment, and inhibiting adipogenesis may enhance haematopoietic recovery in BM transplantation [34]. Boyd et al. showed that AML cells disrupt endogenous myelo-erythropoiesis by compromising the adipocytic niche [35•].

In contrast, Tabe et al. showed that the interaction between acute monocytic leukaemia cells and adipocytes prevented their apoptosis through an increase in fatty acid β-oxidation (FAO) and upregulation of PPARγ, FABP4, CD36 and BCL2 genes. The co-culture increased adiponectin receptor gene expression and its downstream target stress response kinase AMPK. Pharmacological inhibition of FAO induced the integrated stress response mediator ATF4 and apoptosis in monocytic cells, suggesting that disruption of FAO in bone marrow adipocytes may be a potential therapeutic strategy for AML therapy [36]. Lee et al. performed a high-throughput screen and identified avocatin B, a FAO inhibitor, as a novel compound with cytotoxic activity in AML [37]. Ye et al. used a mouse model of blast crisis chronic myeloid leukaemia (CML), where adipose-resident CD36+ LSCs had increased FAO. CD36(+) LSCs and were protected from [38]. Lastly, Shafat et al. showed that BM adipocytes support the survival and proliferation of AML cells in vivo and in vitro. The AML blasts induce phosphorylation of lipase in adipocytes, which consequently activates lipolysis. Co-culturing AML cells with adipocytes upregulates the fatty acid-binding protein-4 (FABP4) messenger RNA. Knockdown of FABP4 reversed the AML cell protection from adipocytes [39].

## Sympathetic Neural Cells

Recent evidence showed that CD34^+^ cells also expressed beta2-adrenergic and dopamine receptors. G-CSF-treatment upregulated the neuronal receptor expression on CD34^+^ cells. Finally, adrenaline and noradrenaline regulated CD34^+^ cell motility and proliferation, in vitro as well as in vivo [40]. Patients with myeloproliferative neoplasms have reduced sympathetic nerve fibres, supporting Schwann cells and Nestin+ MSCs in the bone marrow, mainly due to bone marrow neural damage by interleukin-1β, which is produced by mutant HSCs and favours their expansion. Treatment with β3-adrenergic agonists prevented the loss of Nestin+ MSCs and blocked MPN progression [41]. The sympathetic nervous system (SNS) was shown to promote leukaemic bone marrow infiltration in an MLL-AF9 AML model. AML disrupts SNS nerves and the quiescence of Nestin+ niche cells, leading to an expansion of altered mesenchymal stem and progenitor cells at the expense of HSC-maintaining niche cells [42]. Non-myelinating Schwann cells activate latent TGF-β, which affects HSC maintenance and repopulating activity. Autonomic nerve denervation resulted in rapid loss of HSCs from BM, by reducing the number TGF-β-producing cells [43].

## T Cells

T cells are known to be a crucial part of the immune system’s defence against malignancy; however, there is increasing evidence to show that T cells can be dysregulated in the presence of AML, and their actions can be subverted to a more immune-suppressed state [44]. Here, the effects on the CD8+ cytotoxic cells and multiple subsets of the CD4^+^ T cells are considered. These mechanisms are important, as T cells have been implicated in the development of treatments for AML, for example creating chimeric T cells specifically targeting the tumour, and that T cells have a role in the success of current treatments such as stem cell transplantation [44–47]. T cells require co-stimulatory molecules during activation, and this can be counteracted by upregulation of inhibitory molecules, such as programmed cell death (PD-1), CD244, CD160 and T cell immunoglobulin and mucin- domain containing-3 (TIM-3) [44].

Jia found that CD8^+^ T cells in the bone marrow of AML patients were more terminally differentiated, expressing more factors associated with exhaustion and had reduced function (in killing capacity and cytokine production) compared to those in the peripheral blood [48]. They also found higher levels of PD-1 expression in the CD8^+^ T cells in the bone marrow.

Zhang found that the PD-1 ligand was upregulated on the tumour cells in a murine leukaemia model in vivo. When PD-1 was knocked down, or the mice were given an inhibitory antibody, they had a reduced tumour burden and a larger antileukaemic response [49]. TIM-3, another inhibitory receptor, has also been implicated in T cell exhaustion [50] and is found at higher levels on marrow resident T cells. It was also found that expression of TIM-3 and its partner, Gal-9 (expressed on AML cells), was significantly in higher in patients with treatment failure [51]. In contrast, a review by Lamble et al. discussed the concept of T cell exhaustion and reduced functionality and reported that there is still contradictory evidence surrounding whether it occurs in AML, perhaps due to the high heterogeneity of the disease [44]. For example, Schnorfeil et al. found that while PD-1 was upregulated after relapse, they did not find that this was associated with a functional deficit in the T cells. These tests were performed in peripheral blood [52]. This highlights the need for further, larger studies involving more patients to establish the full mechanism, as immune checkpoints are a potential therapeutic target.

Aside from expression of ligands and receptors, there is accumulating evidence for the involvement of secreted factors. Buggins et al. showed that without physical contact, AML cells could produce a protein which lead to blockade of activation of Th1 cells and their cytokine production, and inhibited activated T cells from entering the cell cycle [53]. Milojkovic showed that this tumour-derived supernatant inhibited apoptosis of both AML and the other haematopoietic stem cells [54]. It has been shown that low arginine levels are associated with T cell exhaustion, and that supplementation reverses this. An enzyme called arginase II, expressed by AML cells, has been implicated [55]. Secretion of galectins [56] and production of reactive oxygen species [57] have also been linked to the AML microenvironment and immune suppression.

In contrast to the potential reduced function of some T cells in the AML microenvironment, T-regulatory (T-reg) cells are a subclass of T cells which are involved in self-tolerance [44]. They have been found to work in conjunction with AML by creating a localized zone where HSPC can reside on the endosteal surface and improve their survival [58]. Studies have shown accumulation of Tregs in the bone marrow in AML [59]. Han et al. showed that AML can express a Treg co-stimulatory ligand ICOS-L, activating Tregs directly, and they can also induce some CD4^+^ cells to become inducible Tregs. These ICOS^+^ T cells can then produce IL-10 which encourages the proliferation of AML in turn [59] and were shown to have an inhibitory action on T effector cells.

Aside from the traditional Th1/Th2 axis, a review by Li et al. concluded that there was evidence of Th17 dysregulation in AML, both in Th17 cells, and in the production of Th17-associated cytokines [60]. More recent papers have concluded similar results, for example Han et al. showed an increase in Th17 cells in peripheral blood, and in the bone marrow in AML compared to healthy controls, and an increase in their associated cytokines [61]. They also found a lower frequency of Th1 cells. Higher levels of Th17 and lower Th1 were associated with a poorer prognosis. Wróbel also found in 2014 that a particular IL-17 polymorphism was associated with an increased susceptibility to AML [62].

In summary, there is evidence of dysregulation of all subsets of T cells in the AML microenvironment, and it appears that AML may have a role in enabling this dysregulation. This involves both expression of both cell-bound and secreted molecules. Given T cells are one of the crucial mechanisms for many AML treatments, further investigation and expansion from murine models may yield further insight.

## Mesenchymal Cells and CXCL12-Abundant Reticular Cells

Mesenchymal stem cells (or stromal cells) can be identified by the expression of Nestin, LepR, Prx-1 or Mx-1 [5]. The perivascular niches include CXCL12-abundant reticular (CAR) cells, Nestin-positive (Nestin^+^) MSCs and leptin receptor-positive (LepR^+^) MSCs. Nestin^+^ MSCs, which usually co-localize with adrenergic nerve fibres, can affect HSC homing to the BM [63]. Stem cell factor (SCF) conditional deletion from LepR^+^ stromal cells reduced HSC number in the BM [64]. During haematopoiesis, the HSC are retained in cellular niches, which are created by stromal cells in order to grow and differentiate [65]. Co-culturing stromal cells with AML blasts was shown to inhibit cytotoxic drug-induced apoptosis via soluble factors as well as cell contact-mediated pathways [66]. Direct contact of AML cells with HS5 (a human stromal cell line) can significantly increase their proliferation, viability and colony formation [67]. When AML cells were cultured on patient-derived multipotent MSCs, their proliferation capacity was shown to be significantly higher compared to when they were cultured on MS5 (mouse stromal cell line) or liquid culture [68••]. Abdul-Aziz et al. investigated the cytokine profile of AML cells cultured in a co-culture system using bone marrow mesenchymal cells. It was shown that macrophage migration inhibitory factor (MIF) was highly expressed by primary AML, and that IL8 was increased in the co-cultures, promoting AML survival [69]. Co-culturing primary human AML cells with normal BM mesenchymal stem cells (MSCs) supported their proliferation and survival in vitro through the release of soluble mediators. This effect was independent of cytogenetic abnormalities [70••].

Studies on the chemokine CXCL12 (also known as stromal derived factor-1 (SDF-1)) revealed that it is highly expressed in a population of reticular cells, the CXCL12 expressing reticular (CAR) cells. They are scattered throughout the bone marrow, creating a network in the perivascular region of BM, either surrounding the sinusoidal endothelial cells or located near the endosteum. CAR cells are progenitors of mesenchymal stromal cells and can differentiate into osteoblasts or adipocytes. These cells have a fundamental role in maintaining of the HSC in bone marrow stromal cell niches [71]. Early B cell factor (Ebf3) is secreted from leptin receptor expressing- CAR cells, which subsequently regulates the interplay between osteogenesis and haematopoiesis [72]. Their differentiating capacity as well as their ability to release high levels of CXCL12 make these cells potential candidates for targeting. CXCL12 and its receptor (CXCR4) have been implicated as critical mediators of the stromal-leukaemic cell interaction, affecting AML cell survival [73], migration [74], adhesion [9, 75] and disease progression by acting as a survival factor for both normal and leukaemic haematopoietic cells. Leukaemic cells use CXCL12/CXCR4 axis to access the protective BM niches, which usually tend to be restricted to normal HSCs. As a result, they reside in a safe environment that promotes their survival and protects them from chemotherapy [6]. Inhibiting the axis can interrupt this communication and induce AML cell mobilization away from the protective niches, where they can be targeted by chemotherapy. Lastly, CXCR4 expression on AML cells has been shown to adversely affect patient survival [76].

## Conclusion

There is a definite clinical need to improve treatment in AML, which remains to be a rapidly progressing leukaemia with poor prognosis and high relapse rate. The BM microenvironment interacts with normal HSC and leukaemic cells in several ways affecting their cellular functions, including their proliferation, differentiation, adhesion, quiescence, trafficking and clonal expansion [77]. The mechanism of stromal-mediated protection of leukaemic cells and their anchorage in the BM is complex and involves many cytokines, chemokines and adhesion molecules. Interactions between the leukaemic cells and other cells in the BM microenvironment are known to be vital for the maintenance and progression of chemotherapy-resistant AML [78], where blasts are thought to remodel the BM niche, favouring leukaemia growth and suppressing normal haematopoiesis through exosome secretion [79••]. As a result, the BM is considered to provide a primary site for minimal residual disease, which causes relapse after chemotherapy and is critical in mediating drug resistance. Given the AML dependence on the BM microenvironment, a better understanding of the biology and interactions between the leukaemic cells and the haematopoietic niche is needed, in order to develop therapies that co-target several signalling pathways simultaneously.
